# Factors Affecting Diet Variation in the Pyrenean Rock Ptarmigan (*Lagopus muta pyrenaica*): Conservation Implications

**DOI:** 10.1371/journal.pone.0148614

**Published:** 2016-02-10

**Authors:** Ricardo García-González, Arantza Aldezabal, Nere Amaia Laskurain, Antoni Margalida, Claude Novoa

**Affiliations:** 1 Instituto Pirenaico de Ecología (CSIC), Jaca, Spain; 2 Plant Biology and Ecology Department, Basque Country University, Bilbao, Spain; 3 Department of Animal Production, Division of Wildlife, Faculty of Life Sciences and Engineering, University of Lleida, Lleida, Spain; 4 Division of Conservation Biology, Institute of Ecology and Evolution, University of Bern, Bern, Switzerland; 5 Office National de la Chasse et de la Faune Sauvage, Prades, France; University of Pretoria, SOUTH AFRICA

## Abstract

The Pyrenean rock ptarmigan (*Lagopus muta pyrenaica*) lives at one of the southernmost limits of the ptarmigan range. Their small population sizes and the impacts of global changes are limiting factors in the conservation of this threatened subspecies. An effective conservation policy requires precise basic knowledge of a species' food and habitat requirements, information that is practically non-existent for this Pyrenean population. Here, we describe the diet of a ptarmigan population in the Eastern Pyrenees, the environmental factors influencing its variability and the relationship between diet floristic composition and quality. Diet composition was determined by microhistological analysis of faeces and diet quality was estimated from free-urate faecal N content. Our results show that grouse diet is based mainly on arctic-alpine shrubs of the Ericaceae family, as well as dwarf willows (*Salix* spp.) and *Dryas octopetala*. The most frequently consumed plant species was *Rhododendron ferrugineum*, but its abundance in the diet was negatively related to the diet nitrogen content. Conversely, the abundance of *Salix* spp., grass leaves and arthropods increased the nitrogen content of the diet. Seasonality associated with snow-melting contributed the most to variability in the Pyrenean ptarmigan diet, differentiating winter from spring/summer diets. The latter was characterised by a high consumption of dwarf willows, flowers, arthropods and tender forb leaves. Geographic area and sex-age class influenced diet variability to a lesser extent. Current temperature increases in the Pyrenees due to global warming may reduce the persistence and surface area of snow-packs where preferred plants for rock ptarmigan usually grow, thus reducing food availability. The high consumption of *Rh*. *ferrugineum* characterised the diet of the Pyrenean population. Given the toxicity of this plant for most herbivores, its potential negative effect on Pyrenean ptarmigan populations should be evaluated.

## Introduction

Diet composition and quality are significant ecological factors that may affect both breeding success and survival of *Lagopus* populations [1,2]. The availability of food for adults prior to breeding has long been thought to play a critical role in the onset of seasonal reproductive events and in particular in egg laying [3, 4]. Knowing the diet composition, its spatio-temporal variations, and the factors influencing diet variations is therefore crucial to understanding the relationships of the species with its environment and to properly adjust species conservation policies [5–7].

The Pyrenean rock ptarmigan (*L*. *m*. *pyrenaica*) dwells in one of the southernmost limits of the species distribution range and their nearest relatives live in the Alps (*L*. *m*. *helvetica*), 400 km away. This species is listed in Annex 1 of the European Bird Directive (Council Directive 79/409/ EEC of 2 April 1979 on the conservation of wild birds), which aims to preserve species and their habitats. Habitat conservation and management requires basic knowledge about the habitat requirements of the species [8]. Their geographical isolation, along with the small population size and low genetic diversity, may increase their vulnerability to global change [9–11].

Information on the diet composition of the Pyrenean ptarmigan is scarce [12–14]. Previous studies showed that the diet is largely composed of ericaceous plants (*Vaccinium* spp., *Arctostaphylos alpina*, *Rhododendrun ferrugineum*, *Calluna vulgaris*) but includes, in some areas such as the Western Pyrenees, other typical alpine species (dwarf willows, *Dryas octopetala*) and a variable proportion of forbs and arthropods in the summer, in agreement with results obtained at higher latitudes [15–17].

Some studies of food habits noted the significance of different factors affecting ptarmigan diet composition. Gelting [18] and Bossert [17] showed seasonality, and snow cover was demonstrated to be one of the most important factors in ptarmigan feeding variation. Other authors [19] indicate sex-age differences as an important source of ptarmigan diet variation. In the Pyrenees, Garcia-Gonzalez et al. [13] and Boudarel & Garcia-Gonzalez [14] showed differences in ptarmigan diets in zones with different geologic substrates, which likely affected vegetation characteristics of feeding grounds in ptarmigan habitats.

Protein content is one of the fundamental components of bird nutrition [20–21], and some authors suggest that it is even more important than metabolizable energy when ptarmigans select food during the winter [17, 22] or the breeding period [23]. We determined free-urate faecal nitrogen in our ptarmigan population, which was used as a proxy of crude protein content and diet quality [24].

In the present study, we describe the diet composition of a ptarmigan population in the Eastern Pyrenees, testing the influence of different intrinsic (sex-age class) and external (zone, season, year, altitude) factors on its variation. We also analyse the relationship of diet components with diet quality, discussing the conservation implications that these results may have in a scenario of global change.

## Material and Methods

### Ethical statement

The study was conducted in full compliance with French and Spanish laws and regulations, including a licence from the Office National de la Chasse et de la Faune Sauvage (ONCFS) and Ordesa y Monte Perdido National Park (PNOMP) for sampling Pyrenean rock ptarmigan faeces and plant species. Radio-tracking of Pyrenean rock ptarmigan was not a part of this study. Radio-monitoring was part of a larger study on demography and habitat use of Pyrenean rock ptarmigan conducted by the ONCFS, for which all permits and ethical and legal requirements were met and enforced. According to the guidance of ONCFS (France) and the Ethics Committee of CSIC (Spain), when no animal experiments are carried out, no other licences are required.

### Study area and grouse population

**Fig 1:**
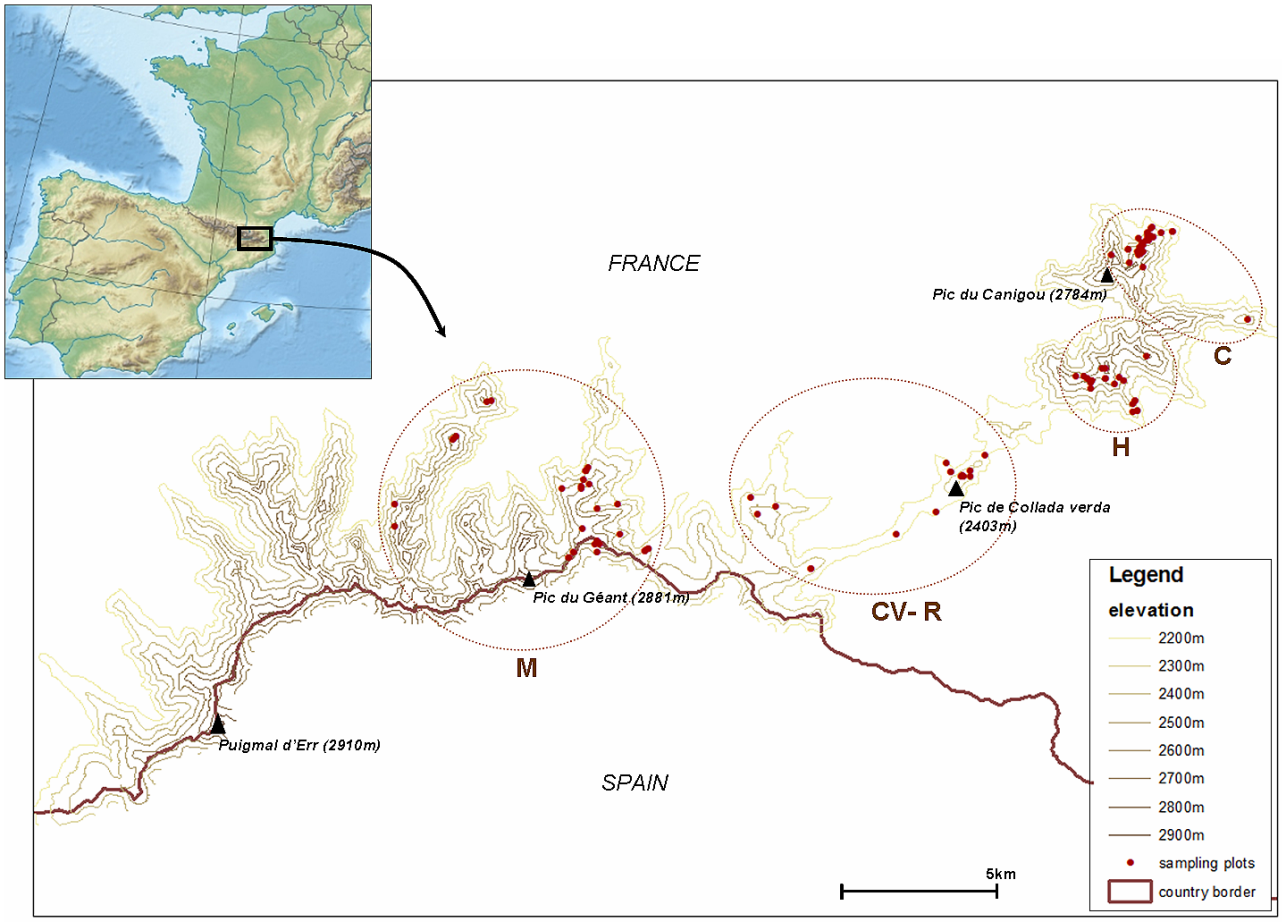
Location of the study area in the eastern French Pyrenees. The map represents the four zones (M = Mantet, CV-R = Collada Verda—Roja, H = Sept Hommes—Tres vents, C = Canigou—Barbet) in which faecal samples were collected. On some occasions, faecal samples were collected in the same sampling plot (i.e. breeding pair, female with chick).

The study was carried out from 2002 to 2004 on the Canigou-Carrança massif (42°31' N, 2°29' E) in an area of 50 km^2^, where a population of Pyrenean rock ptarmigan has been monitored for the last 20 years [25]. This area represents the eastern limit of the distribution of rock ptarmigan in the French Pyrenees and the southern limit in Western Europe (Fig 1). The habitat occupied by the species is mainly located at altitudes ranging from 2200 m to 2800 m with a preference for northern and eastern exposures [26]. The vegetation at the lowest elevations is dominated by open woodlands of Mountain Pine *Pinus uncinata* associated with ericaceous shrublands (*Rhododendron ferrugineum*, *Vaccinium myrtillus*, *V*. *uliginosum*, *Empetrum nigrum*). The upper parts of the habitat are characterised by sparse alpine vegetation (*Dryas octopetala*, *Salix herbacea*, *Silene acaulis*, *Saxifraga* sp.) [27, 28] interspersed with large expanses of gneiss screes. At 2150 m, mean annual temperature is 4.6°C and mean annual precipitation exceeds 1400 mm (data from Meteo France–meteorological station at the Cortalets Refuge, the closest (12 km) weather station to the study area). The seasonal distribution of rain shows a high inter-annual variability, but summer is generally the driest season. From 2000 to 2009, the date of total snowmelt varied between 30 April and 18 June [7].

The main agricultural activity is cattle grazing, which takes place from early June to late October, with moderate stocking rates of 0.05–0.10 animal units/month/ha.

### Droppings collection and microhistological analysis

From 2002 to 2004, 121 faecal samples were collected during radio-tracking surveys and summer counts of the Canigou-Carança rock ptarmigan population [25]. Droppings were collected usually after seeing the birds, so we were therefore able to collect fresh samples (which is important for temporal allocation of diets), and to identify sex-age class for most of the samples. Faecal samples of young birds were identified by their size. A faecal sample was composed of several droppings or pellets (5 to 20).

Faecal samples come from four different zones: Canigou-Barbet (33%), Mantet (28%), Sept Hommes-Tres Vents (23%), Collada Verda-Roja (16%) (Fig 1). For each sample, date, location, altitude and sex-age class were recorded. Seasonally, 39% of the samples correspond to the spring, 32% to the summer and 29% to the winter. Season definition was adapted to climatic and phenological conditions of the Pyrenean rock ptarmigan habitat. Spring corresponds to a time of snow melting and onset of plant growth (May–June), summer to a climatically favourable period with abundant food resources (July to September), and winter to unfavourable climatic conditions and scarcity of food resources (October to April). Sixty-seven percent of samples correspond to post-melting (snow-free) periods and 33% to pre-melting periods, the latter mainly from the winter season but also from spring in years of late snowmelt (S2 Table).

Droppings were preserved in aceto-formalin and stored until analysis. When possible, we took 2 ml of droppings from each individual sample. Faecal samples were treated with 1 ml nitric acid, rinsed in 200 ml boiling water and passed through 1 and 0.2 mm sieves. The intermediate fraction was collected and stored in 4 ml aceto-formalin until microscopic analysis [29]. Some samples were so small that 2 ml were not available for analysis. In these cases, we used the entire sample available. We had previously confirmed that the small volume used for these samples should not have affected the number of identified items (S1 Fig).

A portion of prepared droppings was applied to a microscopic slide and observed under 100x-400x magnification [13]. Diet content was determined by microscopically identifying leaf, seed, fruit, woody elements and arthropod fragments from a reference collection of possible grouse food species in the study area (available at the Pyrenean Institute of Ecology-CSIC, Jaca, Spain). On microscopic slides, continuous transects were surveyed identifying all fragments encountered in the microscopic field [30]. An average of 168 (100–364) identified items per faecal sample was achieved. Relative frequencies of dietary items were calculated dividing the number of identified fragments of each species by the total number of identified fragments.

Typically, only the leaf epidermis has recognisable characters to identify microscopic fragments at the species or genus level [31]. Flower, seed, fruit or woody elements are normally identified at higher taxonomic levels. Nevertheless, due to a detailed reference collection and technician training we were able to identify these elements for the main species of ptarmigan diet. In addition to taxonomic identification, food type classification of microscopic items is relevant because of the differences in nutritional value [24, 32]. Flowers, seeds and fruits normally provide larger quantities of protein, lipids, vitamins and minerals than leafy or stem materials. Stems and buds have high levels of lignin content and therefore a lower digestibility. The nutritional value of leaves varies to a greater extent with their phenological state [33].

### Diet quality

N excretion (total N) is related to N intake [24], but the total N of bird droppings is composed of faecal N and urinary N. Faecal N (N alfa-amino) derives mostly from protein metabolism. Urinary N is composed of waste products like uric acid and ammonium salts. Furthermore, it can contain other products like ornithuronic acid but in principle contains mostly water-solubles [34]. Faecal and urinary N must be separated to obtain a better estimation of the consumed digestible protein than using total N only. To liberate the droppings from the urine portion and to keep the faecal portion only, we washed them with formaldehyde [35]. Faecal samples were added to 10 ml acetic-formalin and recipients were shaken in an ultrasonic bath for one hour. We determined the N content of droppings (Kjeldahl method) before (total N) and after washing (free-urate faecal N), and considered the N content (% DM) of droppings after washing as a proxy of digestible protein ingested [36] and hence, of diet quality. In samples that could not be washed due to small quantities, we estimated free-urate faecal N from the following linear regression: [free-urate faecal N] = 0.518 [total faecal N]– 0.528 (r^2^ = 0.705; n = 74).

### Statistical analysis

The relative importance of different factors (year, season, zone, altitude, and sex-age) on the botanical composition of diet was evaluated using constrained (canonical) ordinations [37, 38]. For canonical ordination analysis only droppings from individuals of known sex-age class were considered. Fourty-nine female, 35 male and 13 juvenile faecal samples were used for these analyses; for all other analyses we used all 121 samples.

We defined the diet composition data as the response matrix, with 97 samples and 16 plant categories grouping all taxonomic categories into 16 taxonomically and nutritionally relevant food variables (for a definition see S2 Table). The response matrix was composed of species abundance in percentage vs. individual ptarmigan (log-transformed, hereafter *diet matrix*). As a first step, to select the appropriate constrained ordination technique for hypothesis testing, *diet matrix* was subjected to a detrended correspondence analysis (DCA), detrending by segments and with non-linear rescaling of the axes. Since the length of the extracted gradients was higher than three standard deviation units (3.379 uSD), we assumed a unimodal response and conducted a Canonical Correspondence Analysis (hereafter CCA) following the recommendations of Ter Braak [37].

We created five constraining (explanatory) matrices: (1) *year matrix*, formed by three dummy variables (2002, 2003 and 2004); (2) *season matrix*, in which three dummy variables were defined (“spring” which includes samples collected from May to June, “summer” including samples from July to September, “autumn-winter” including samples from October to April); (3) *zone matrix*, with four dummy variables (Canigou-Barbet, Mantet, Sept Hommes-Tres Vents, Collada Verda-Roja); (4) *altitude matrix*, with one continuous quantitative variable; (5) *sex-age matrix*, with three dummy variables (male, female and juvenile). The null hypothesis was that *diet matrix* is independent of the five explanatory matrices. To avoid multicollinearity problems, one dummy variable per set was released. The collinearity was tested for each explanatory matrix using Variance Inflation Factor (VIF), following Petraitis et al. [39]. The total variation explained (TVE) was calculated as the relationship between the trace and the sum of all canonical extracted axes [40]. A Monte Carlo permutation test was performed to determine the accuracy of the relationship (999 randomizations) between the response and explanatory matrices. The sum of all canonical eigenvalues was used to build the F-ratio statistic [37]. Only when p<0.05, adjusted for multiple comparisons by the Bonferroni correction [38], was the relationship between the two data sets considered significant. If the CCA model was significant, a forward stepwise procedure was carried out to select a reduced model including only significant variables for each matrix. Improvement of the reduced model with each new selected variable was determined by a Monte Carlo permutation test with 999 randomizations. Finally, we conducted a variance partitioning analysis performing partial CCAs to evaluate the relative importance of a given explanatory matrix after controlling the variation caused by the rest of the matrices defined as covariables [38, 40]. Every partial model was tested by the Monte Carlo permutation test. All the analyses were conducted using CANOCO for Windows v. 4.5 [41].

To explore the relationship of diet components with diet quality, Pearson correlations were calculated between the free-urate faecal nitrogen and the relative abundance of main diet components. These correlations were tested with the SPSS 15.0 version package.

## Results

### Botanical composition of diets

The rock ptarmigan diet in the Pyrenees was quite diverse based on the ninety-eight different items identified in the microhistological analysis of faecal samples (S1 Table). Considering large food type categories, leaves (52 ± 3%, mean ± standard error) and stems and buds of woody species (22 ± 2%) were the most important components of the diet on an annual basis (mean of three years). Leaves corresponded almost completely to dicotyledonous plants (herbaceous or woody), while grasses were scarce in the ptarmigan diet. Other important foods were flowers (17 ± 2%), arthropods (3 ± 1%), and seeds and fruits (6 ± 1%). In respect to plant species, *Rhododendron ferrugineum* (21 ± 3%), other Ericaceae (9 ± 2%) such as *Calluna vulgaris*, *Vaccinium myrtillus*, *V*. *uliginosum*, alpine dwarf willows (*Salix herbacea* and *S*. *pyrenaica*, 10 ± 2%), herbaceous Asteraceae (*Crepis pyrenaica*, *Leucanthemopsis alpin*a, *Leontodon* sp., 16 ± 2%) and *Jasione crispa* (5 ± 1%) were the most abundant species in ptarmigan droppings (S1 Table).

**Fig 2:**
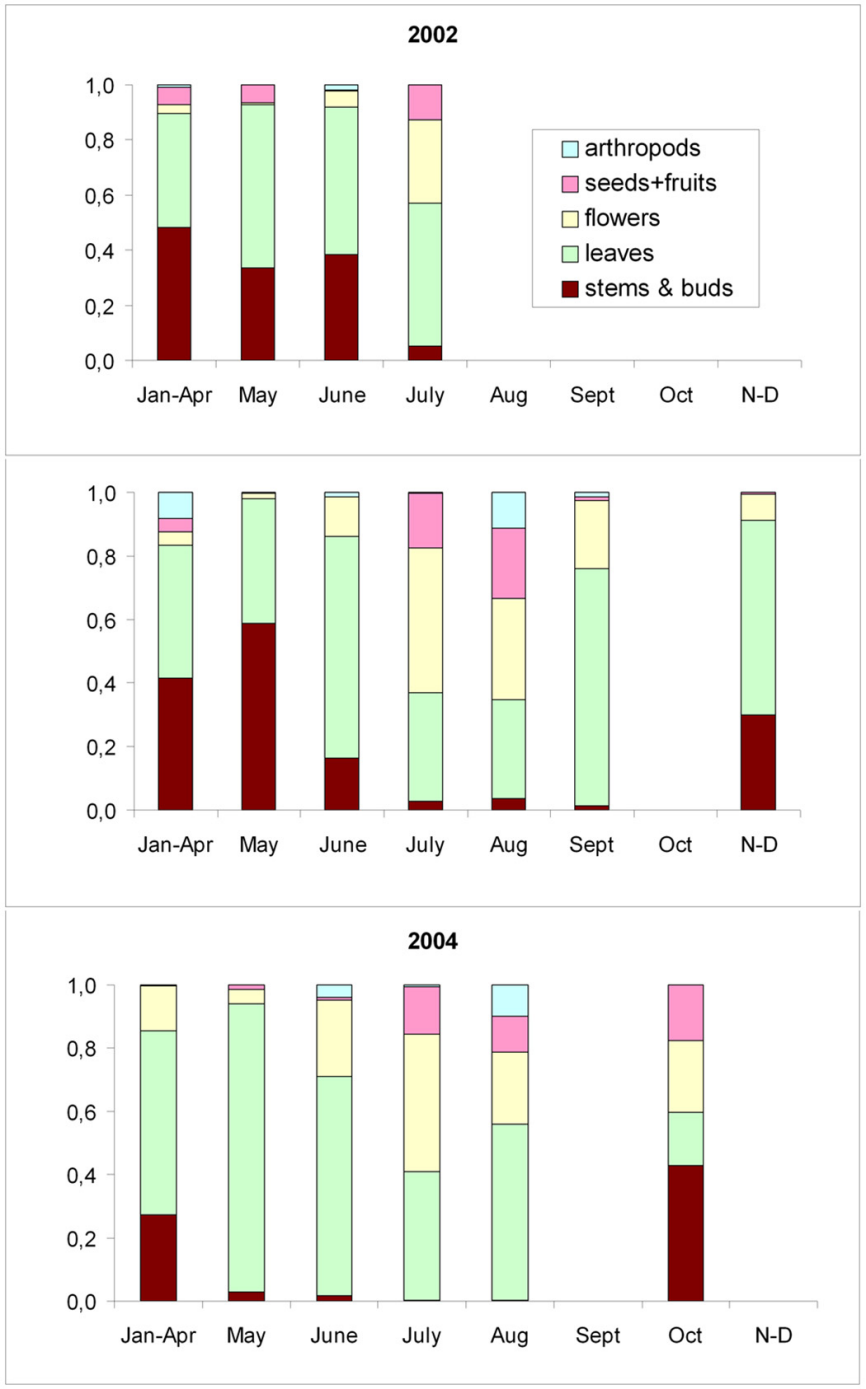
Monthly/seasonal variation of Pyrenean ptarmigan diets in the sampled years grouped by simplified morphological categories. Autumn (O-D) and winter (J-A) months have been grouped.

Interannual and seasonal diet variations were observed (Fig 2). In winter, leaf epidermis and woody elements (stems and buds) formed the bulk of the diet in approximately equal parts. Woody species predominated (*Rhododendron ferrugineum*, *Calluna vulgaris*, *Salix* spp., *Vaccinium* spp., *Dryas octopetala*, *Pinus uncinata*). Forbs such as *Oxytropis foucadii*, *Alchemilla plicatula*, *Cerastium fontanum* and *Jasione crispa*, were also important dietary components in some years. This type of diet was maintained until mid-June in 2002 and 2003, but changed in April in 2004 (Fig 2). Between winter and summer (June), a transition diet occurred. At this time, stems and buds decreased in proportion and leaves correspondingly increased. The main species were *Calluna vulgaris*, *Salix* sp., *Oxytropis foucadii* and *Alchemilla plicatula*. This type of diet lasted a few weeks in 2002 and 2003, but was longer in 2004 (from April to mid-June). In summer (July-September), flower elements predominated and an important contribution of seeds, fruits and arthropods was observed. The main species belonged to the Asteraceae (*Leucanthemopsis* sp., *Leontodon* sp., *Hieracium* sp.) and Campanulaceae (*Jasione crispa*) botanical families. *Saxifraga moschata*, *Salix* sp. and *Vaccinium* sp. were also important components of the summer diet (S1 Table).

### Factors affecting diet composition

**Fig 3:**
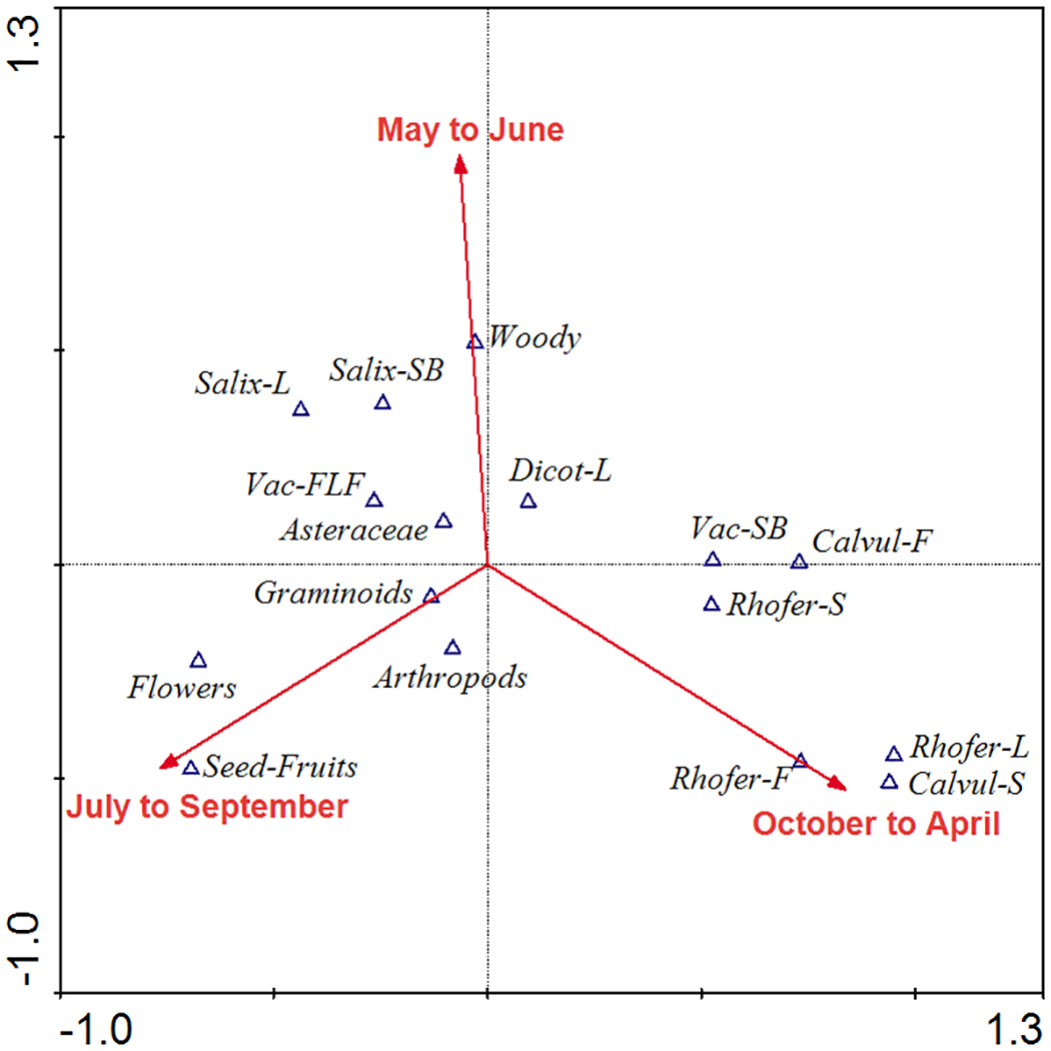
Partial CCA ordination diagram (with *season* as explicative matrix, and *zone* as covariable matrix), in which significant seasons are represented as vectors. Abbreviations for plant categories: Calvul = *Calluna vulgaris*; Rhofer = *Rhododendron ferrugineum*; Salix = *Salix* spp.; Vac = *Vaccinium* spp.; L = Leaves; F = Flowers; Fr = Fruits; S = Stem; B = Buds.

All constraining matrices significantly explained some of the variability of the ptarmigan diet, but in small proportion (Table 1). The *season* matrix showed the highest TVE (13.8%), followed by *zone* (reduced model 7.8%), *altitude* (3.7%), *sex-age* (reduced model 2.6%) and year (reduced model 2.6%). In relation to seasons, autumn-winter diets were associated with *Rhododendron ferrugineum* and *Calluna vulgaris*, whereas summer diets were related to the consumption of flower and fruits parts of plants. Spring diets showed the most variability with a dominance of *Salix* ssp., *Vaccinium* ssp., other woody species and dicotyledonous leaves (Fig 3). On the other hand, diets from Canigou-Barbet were the most different explaining a significant part of diet variability. Altitude, year and sex-age explained a small part of diet variation. Males and females did not differ in diet composition (Table 1).

**Table 1:** Forward stepwise CCA and reduced models for the five explanatory (constraining) matrices in which the response matrix is *diet matrix* (i.e., botanical composition of ptarmigan diet). TVE% (*x*) = total variation explained in percentage (TVE% of reduced model); λ_1_, λ_2_, λ_3_ are the eigenvalues of the first three axes. Trace is the sum of all canonical eigenvalues. Only significant variables (p<0.1) have been indicated. The significant TVE% is showed in bold. In the case of dummy variables, one level (category) was deleted to avoid collinearity problems when fitting the model: *May to June* (in season matrix), *Collada Verda-Roja* (in zone matrix), *juvenile* (in sex-age matrix), *2002* (in year matrix).

Constraining matrix	TVE%	Reduced model	λ_1_	λ_2_	λ_3_	F-ratio	p	Trace
*Season*	**13.8**	Full model	0.359	0.123	0.549	7.55	0.001	0.48
		*July to September*				9.58	0.001	0.32
		*October to April*				5.11	0.001	0.16
*Zone*	10.4 (**7.8**)	Full model	0.275	0.056	0.028	3.58	0.001	0.36
		*Canigou-Barbet*				8.07	0.001	0.27
*Altitude*	**3.7**	*Altitude*	0.130	0.605	0.517	3.69	0.001	0.13
*Sex-age*	4.1 (**2.6**)	Full model	0.127	0.017	0.566	2.02	0.008	0.14
		*Female*				2.40	0.009	0.09
		*Male*				1.61	0.097	0.05
*Year*	3.3 (**2.6**)	Full model	0.095	0.019	0.610	1.60	0.06	0.11
		*2004*				2.41	0.012	0.09

Partial CCA analyses indicated that *season* (as an explicative matrix) and *zone* (as a covariable matrix) together significantly explained the highest TVE (20.1%, sharing 1.5% of variation), but 79.9% of residual variation remained unexplained. The remaining combinations explained less than 16.5% of TVE, and no fractions of variation were shared between explicative and covariable matrices, except for altitude, which shared 2.2% of variation with season.

### Relationships between diet components and diet quality

We tested linear correlations between the main groups of components of the ptarmigan diet determined by microhistological analysis and free-urate faecal N of each faecal sample. Free-urate faecal N, and therefore diet quality, was positively associated with the proportion of arthropods (r = 0.565, p < 0.001, n = 35), graminoid leaves (r = 0.276, p < 0.02, n = 66) and *Salix* spp. (r = 0.529, p < 0.001, n = 34) in particular with *Salix pyrenaica* (r = 0.652, p < 0.01, n = 14), or *Dryas/Salix* (r = 0.709, p < 0.02, n = 10), which includes undifferentiated elements of these two taxa. On the contrary, free-urate faecal N was negatively correlated with the proportion of *Rhododendron ferrugineum* (floral element: r = -0.370, p < 0.05, n = 29; bud: r = -0.373, p < 0.01, n = 43; leaves: (r = -0.380, p < 0.04, n = 29), *Pinus uncinata* leaves (r = -0.80, p < 0.03, n = 7 and *Calluna vulgaris* (r = -0.436, p < 0.03, n = 24). The sum of all woody elements (stems and buds of different species) also showed a negative trend with diet quality (r = -0.201, p = 0.084, n = 75).

## Discussion

### Diet characteristics

Current rock ptarmigan distribution in the Holarctic region is a typical example of an arctic-alpine disjunction. This assumes that during glacial retreat at the end of the Pleistocene, plants and animals typical of cold steppes moved northward or ascended in altitude in southern massifs looking for similar cold climatic conditions [42]. The diet of the ptarmigan in the Pyrenees consists of many plant species (or their arctic-alpine vicariants) also found in the diet of other populations of ptarmigan at higher latitudes [15, 18, 43] or on mountains with important alpine zones [17, 34, 44, 45]. A large part of the diet consists of species of the Ericaceae family (*Calluna vulgaris*, *Vaccinium* spp., *Loiseleuria procumbes*), *Dryas octopetala* and dwarf willows (*Salix* sp.), which also have an arctic-alpine distribution. A distinguishing feature of the diet in the Pyrenees is the low consumption of *Polygonum viviparum* whose bulbils have high protein content [18, 34] and are considered an essential element in chick growth [43]. The very low consumption of this element in the population of Canigou (S1 Table) could be explained by their scarcity in the study zone and in general in the Pyrenees [46], whose Mediterranean-alpine character may disfavour the abundance of this species typical of long-standing snow-patches.

Another important differential element in the ptarmigan diet in the Eastern Pyrenees is the high consumption of *Rhododendron ferrugineum* (21% of total faecal items). *Rh*. *ferrugineum* is a subalpine shrub (1600–2600 m) abundant on the acidic substrates of the eastern and middle part of the Pyrenees [46]. Its relatively large height (0.3–1.5 m) allows the shrub to protrude above the snow surface and be eaten by rock ptarmigan [47]. In snowy winters it can be an abundant source of food and refuge and this is likely why it is often consumed by rock ptarmigan in the winter in the eastern Pyrenees. Apart from the Pyrenees, its consumption has only been recorded in the Alps, but in less appreciable quantities (6–8%, [17]). This ericaceous plant is distributed throughout the mountains of southern Europe and is lacking in arctic environments. Its distribution is not the result of the arctic-alpine disjunction [48] and it is possible that the coexistence of the Pyrenean ptarmigan with this species has not been as long as with the other arctic-alpine plants on which it feeds. Grouse tend to look for protein-rich foods and avoid those with anti-herbivore compounds [21]. *Rh*. *ferrugineum* contains various alkaloids whose toxicity may be notably detrimental to small animals [49, 50]. Although the Pyrenean rock ptarmigan could have developed detoxifying mechanisms to prevent poisoning by *Rhododendron*, a prolonged consumption of this item in early spring could affect the body condition of hens during the pre-laying period, through the negative effects of phytochemicals on plant digestibility [22, 51, 52]. Our results suggest that an increase of *Rh*. *ferrugineum* in the diet is associated with a drop in diet N content. The negative effects of phytochemicals on grouse nutrition and their possible increase given projected climate change [53] should be investigated for the rhododendron-rock ptarmigan relationship.

### Temporal and spatial variability of ptarmigan diets in the Eastern Pyrenees

The present study shows that seasonal variability is the main factor affecting ptarmigan diet in the Canigou Massif. In the Pyrenees (as usually happens in arctic-alpine environments) the spring season is largely dependent on snow melt and data on snow-melting show inter-annual variations. For that reason, spring diets are the most variable. For instance, snow had melted on average as of 31 May in 2002, as of 5 May in 2003 and as of 10 June in 2004. Thus, in 2003 (earlier snow-melting) spring began one month earlier than in 2002 and 2004. This may explain why the ptarmigan diet in the spring of 2003 differed significantly with respect to the springs of 2002 and 2004 (Figs 2 and 3) while autumn-winter diets were very similar. These results agree with ptarmigan diet studies over several years and seasons [14, 17, 19, 45]. The harsh climatic conditions in the ptarmigan habitat imply strong dietary differences related to seasonal and inter-annual variations of snow cover in arctic-alpine environments [54, 55]. During the pre-melting period, the diet was mainly based on old leaves and buds of dwarf shrubs standing above snow level (Fig 2). The most abundant species in 2002 and 2003 was *Rh*. *ferrugineum*, with *Calluna vulgaris* also prevalent in 2004, both species of low nutritional value [56]. When snow melts, plants and invertebrates that provide food to ptarmigan during the reproductive period begin to grow and several studies have pointed to a close relationship between the timing of snowmelt and reproductive success [4, 25, 57, 58].

During winter months (January to April), conspicuous consumption of items that should not be active during this period (flowers, fruits and arthropods) was indicated by Pyrenean diet analysis (Fig 2 and S1 Table). Flowers and fruits were likely due to dry elements (from the past growing period) preserved by the low temperatures of winter [59]. Arthropods may have been lethargic and incidentally found by ptarmigan under rocks or tree bark, or active and driven by convection currents to high mountain levels, attracted by the snow patches and consumed by alpine birds [60]. The consumption of these scarce elements in winter would indicate a selective trophic behaviour toward high quality food bygrouses.

The spatial influence on diet variations could be attributed to differences in the availability of food resources often linked to snow cover [17, 18]. A significant influence of altitude must be interpreted as a variant explanation of spatial (zone) and temporal (season) factors (e.g. snow cover takes place earlier at low compared to high altitudes or may vary between years in the same location). Short migrations by rock ptarmigan took place seasonally as they searched for better food resources in the high alpine pastures in summer and for climatic refuges in winter at the lowest altitudes [61].

Another cause of spatial diet variation is linked to geological substrate, which often determines quality and diversity of ptarmigan diets [52]. García-González et al. [13] conducted a ptarmigan diet study in the Western Pyrenees and found an almost monospecific winter diet based on *Dryas octopetala* (92%) in basic substrates, and a much more diverse diet based on acidic substrates. This was interpreted as a reduction of trophic niche when a highly preferred food source is found in a situation of resource scarcity (winter). In mixed substrates, such as in the Canigou Massif, the ptarmigan diet was composed of calcifugous plants (*Rh*. *ferrugineum*, *Calluna vulgaris*) as well as calcareous plants (*Dryas octopetala*, *Salix pyrenaica*). Diets in Greenland [18] and Iceland [19] were much more diverse and rich than in Scotland [1] where diets were based mainly on *Calluna vulgaris*, a very poor food plant typical of acidic substrates.

Sex-age class explained a minor part of ptarmigan diet variation in our study (2.6%, reduced model in Table 1). Pulliainen & Tunkari [59] did not find sex-based differences in willow grouse (*L*. *lagopus*) diets in Finland, while Elson et al. [62] found sex-based diet differences associated with habitat use differences between sexes. Some studies point to a significant difference between age classes. Chicks normally eat large quantities of high-protein foods such as arthropods [63] or *Polygonum viviparum* bulbils [43]. Nevertheless, Allens & Clarke [64] found that ptarmigan hens chose foraging patches where high quality plant species were abundant and called their chicks to these foods, which dominated their diet. This is a mechanism to enhance the diet quality of chicks and likely leads to a high similarity between the diets of hens and young birds.

### Relationships between diet components and diet quality

We found free-urate faecal N (which is associated with dietary protein content) to be positively correlated with the consumption of arthropods, graminoid leaves, and dwarf willows in ptarmigan droppings. On the contrary, free-faecal N was negatively associated with the proportion of *Rh*. *ferrugineum* (all parts), *Calluna vulgaris* leaves and the sum of woody elements, which normally occur in winter. Arthropods and dwarf willows are high-quality food for grouse and their consumption increases in the breeding season when available [15, 18, 43]. In contrast, woody elements have high lignin content and are poorly digestible [51]. *Rh*. *ferrugineum* consists of over 30% lignin content in May (unpublished data) and *Calluna vulgaris* has high levels of tannins [34, 65]. These compounds are known to decrease digestibility and may decrease the assimilation of proteins [52].

Our results show that the highest faecal N is related to diet components consumed in spring. The proportion of *Salix* spp., graminoid leaves and arthropods appears to most improve the quality of ptarmigan diets. Leaves of *Salix* spp. are not available until snow melts since they are deciduous species. New graminoid leaves and arthropods are normally not available until after higher temperatures are reached in July and August. As a result, a delay in snow thaw can delay the availability of these richer food items to ptarmigan at the beginning of the laying period [58].

### Conservation implications

We provide for the first time a detailed spectrum of the ptarmigan diet in the Eastern Pyrenees. A precise knowledge of the diet and its seasonal variations can help to detect potential trophic competition with other alpine vertebrate herbivores sharing the same territory. Based on current knowledge, we can infer an absence of overlap with the diets of other Pyrenean alpine herbivorous birds [30] or mammals [66–68].

Our results suggest that Pyrenean rock ptarmigan and the majority of plants on which they feed are the consequence of the arctic-alpine disjunction. This long-standing coexistence between ptarmigan and their primary feeding plants likely implies adaptations in ptarmigan to this type of food, e.g. consumption of plants with low alkaloid content, a general characteristic of the Arctic and Antarctic plants [69]. The substantial incorporation of species with anti-nutritional properties (low concentrations of protein, high levels of alkaloids) may be a handicap to the ptarmigan, on top of other potential threats [6].

The arctic-alpine plants on which Pyrenean rock ptarmigan normally feed usually grow in long-term snowpacks. Current temperature increases in the Pyrenees due to global warming could reduce the persistence and surface of these snowpacks [70] reducing the availability of preferred plants for rock ptarmigan.

The upward altitudinal shift of the subalpine forest in the Pyrenees [71], partially related to the abandonment of pastoral practices, may favour the expansion of *Rh*. *ferrugineum*. This species tends to grow in nearly monospecific communities, forming large colonies in the Pyrenean subalpine belt reducing habitat fragmentation and diversity [72]. As we previously mentioned, *Rh*. *ferrugineum* may be nutritionally disadvantageous for ptarmigan, as its high consumption in winter is associated with a decreased level of protein intake. This adverse effect may be extended in late springs. As low body condition before the pre-breeding period can result in a decrease of reproductive success [3], species management policies should monitor shrub encroachment processes in acidic substrates of the Pyrenees for species such as *Rh*. *ferrugineum*. In addition, stress by humans on wildlife from continuous development of outdoor recreational activities is of increasing concern for biodiversity conservation [73]. Taking into account the poor energetic nutrients provided by *Rh*. *ferrugineum*, winter disturbance related to human activities should be reduced through regulations in the habitat occupied by the species.

We found that Pyrenean rock ptarmigan experiences a drastic seasonal shift from a diet based on shrubs of low nutritional value in winter to a high-quality diet in the spring-summer breeding season. This quality additionally increases with the consumption of flowers and arthropods. This contrasting change in diet begins with the snowmelt, which makes available the new plant and animal resources in the spring. A delay in the date of snowmelt can negatively influence the reproductive success of ptarmigan [25, 55, 74]. Hence, snowmelt likely determines the habitat quality of Pyrenean rock ptarmigan in spring.

## Supporting Information

S1 Fig Relationship between the sample volume used for faecal analysis and the number of identified items. (TIF)

S1 Table Mean abundance (%) of food items recorded by microhistological analysis in Ptarmigan faeces by year and in the total three years. W woody element (stem or bud), L leaf epidermis, F floral element, S fruit, seed or fungus, A arthropod. Second and third horizontal lines mark the items that sum 80% and 90% of the total diet, respectively. Sample size: n = 121; identified items = 20,301.
(PDF)

S2 Table Proportion of the different plant categories and arthropods in all faecal samples analysed. Plant categories are a combination of both taxonomical and morphological types with nutritional significance. Factor value used for the CCA analyses has been noted by each sample. Samples with undetermined sex-age class or zone (marked in yellow) have been suppressed for CCA analysis.
(XLS)
