# Supplementary figures and images for: Factors Affecting Diet Variation in the Pyrenean Rock Ptarmigan (Lagopus muta pyrenaica): Conservation Implications
Source: PLoS One. 2016 Feb 10;11(2):e0148614. doi: 10.1371/journal.pone.0148614 (PMC4749312; doi:10.1371/journal.pone.0148614)

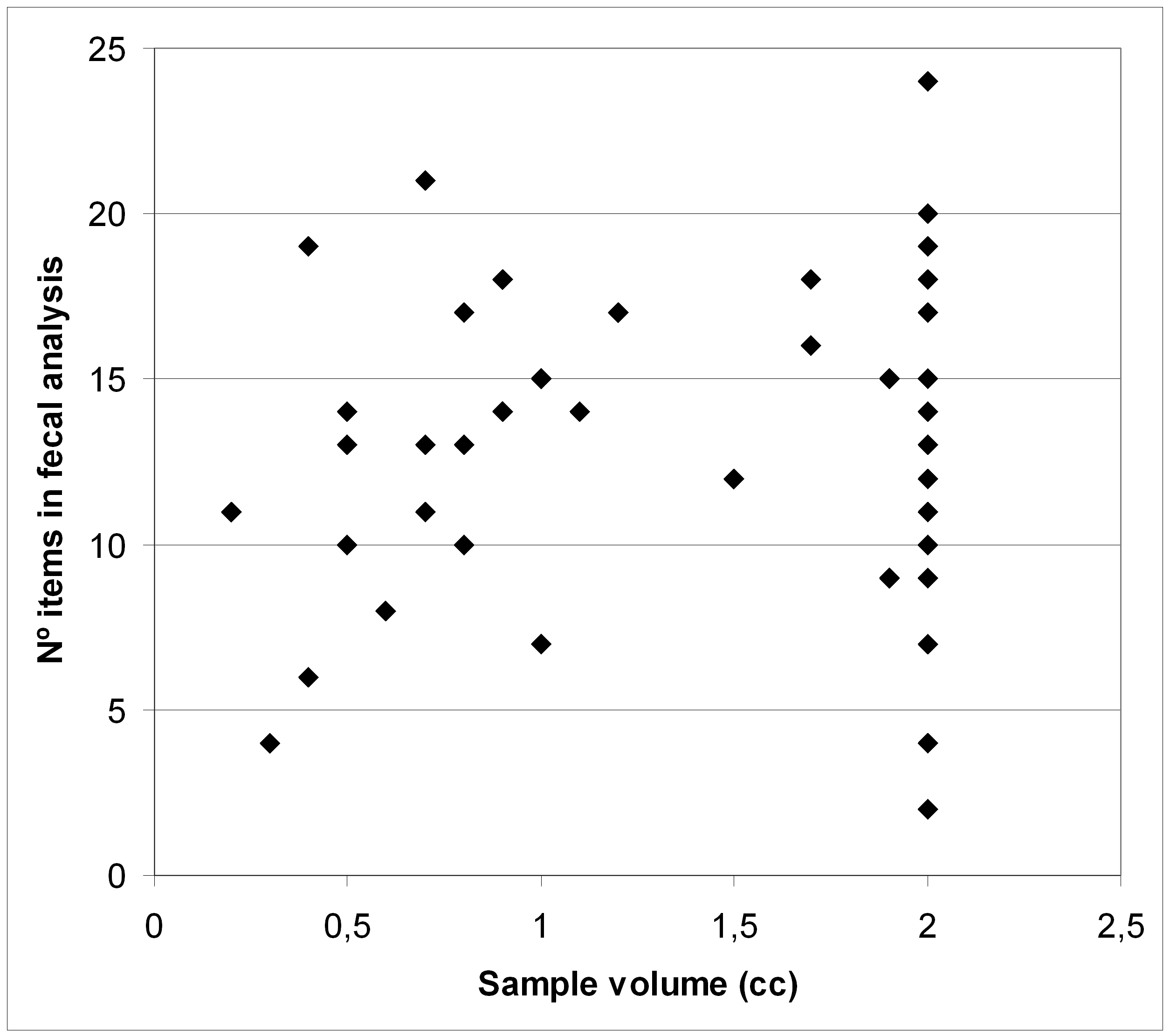

Supplement: S1 Fig — (TIF) [file pone.0148614.s001.tif]
