# Supplementary material for: Factors Affecting Diet Variation in the Pyrenean Rock Ptarmigan (Lagopus muta pyrenaica): Conservation Implications
Source: PLoS One. 2016 Feb 10;11(2):e0148614. doi: 10.1371/journal.pone.0148614 (PMC4749312; doi:10.1371/journal.pone.0148614)
Supplement: S1 Table — W woody element (stem or bud), L leaf epidermis, F floral element, S fruit, seed or fungus, A arthropod. Second and third horizontal lines mark the items that sum 80% and 90% of the total diet, respectively. Sample size: n = 121; identified items = 20,301. (PDF) [file pone.0148614.s002.pdf]

Item	Type	2002	2003	2004	Total
Rhododendron ferrug. bud	W	7,7	10,6	6,3	8,7
Rhododendron ferr. stem	W	12,8	8,7	0,2	7,2
indeterminate flower item	F	1,5	8,5	4,1	5,5
Asteraceae	F	6,1	3,0	8,0	5,2
Jasione crispa	L	2,0	6,6	4,5	4,9
Salix herbacea	L	7,3	3,8	3,9	4,7
Salix pyrenaica	L	6,6	4,7	0,0	3,8
Calluna vulgaris	L	2,2	1,5	8,7	3,8
Alchemilla catalaunica	L	2,8	1,8	6,8	3,5
Arthropod	A	1,0	3,4	2,5	2,6
indeterminate stem	W	6,1	1,9	0,7	2,5
Oxytropis foucadii	L	2,5	0,7	5,2	2,5
Rhododendron ferrug.	L	2,3	2,2	2,7	2,4
Campanulaceae	L	0,1	3,7	2,1	2,3
Saxifraga moschata	L	0,1	3,0	3,0	2,3
Leontodon sp. (vilane)	S	1,0	3,0	1,9	2,2
Rhododendron fer flower	F	1,0	1,7	3,7	2,1
Cerastium fontanum	L	3,3	2,3	0,6	2,1
Crepis pyrenaica	L	5,3	0,2	1,9	1,9
Vaccinium myrtillus (stem)	W	2,6	1,0	1,2	1,4
Hieracium lactucella	L	0,1	1,9	1,7	1,4
Leucanthemopsis alpina	L	0,0	1,8	1,6	1,3
Asteraceae (vilane)	S	2,0	0,1	2,6	1,3
Anthyllis vulneraria	L	4,8	0,0	0,0	1,1
Leontodon sp.	F	0,0	1,3	1,7	1,1
Asteraceae leaf	L	0,0	1,2	1,9	1,1
Vaccinium sp.	L	1,5	1,5	0,0	1,1
Indeterminate bud	W	0,3	2,0	0,0	1,0
Pinus uncinata	L	3,0	0,5	0,2	1,0
Dryas octopetala	L	0,8	1,2	0,9	1,0
Dryas/Salix	L	1,3	1,3	0,0	1,0
Armeria maritima	L	0,1	1,8	0,1	0,9
Vaccinium sp.	S	1,4	0,7	0,8	0,9
Indeterminate fruit	S	1,5	1,0	0,2	0,9
Graminoid	L	1,1	0,5	0,8	0,7
Arenaria grandiflora	L	0,6	0,7	0,8	0,7
Cyperaceae	F	0,0	0,1	2,2	0,7
Indeterminate leave	L	0,9	0,9	6.4	2,5
Vaccinium myrtillus	L	0,0	1,4	0,0	0,7
Vaccinium uliginosum	L	0,0	1,3	0,0	0,6
Salix sp. stem/bud	W	0,6	0,7	0,2	0,5
Armeria alpina	F	0,4	0,5	0,5	0,5
Vaccinium sp. (anther)	F	1,0	0,2	0,2	0,4
Loiseleuria procumbens	L	0,0	0,7	0,0	0,3
Gramineae	F	0,0	0,0	1,1	0,3
Leontodon pyrenaicus	L	0,3	0,3	0,3	0,3
Pinus uncinata stem	W	1,2	0,0	0,0	0,3
Rhododendron ferrug. fruit	S	0,0	0,5	0,1	0,3
Monocot. non graminoid	L	0,0	0,1	0,7	0,3
Pilosella breviscapa	L	0,0	0,0	0,9	0,2
Festuca eskia	L	0,5	0,2	0,2	0,2
Cerastium fontanum	F	0,0	0,1	0,6	0,2
Festuca pyrenaica	L	0,0	0,1	0,7	0,2
Cerastium fontanum seed	S	0,0	0,3	0,2	0,2
Festuca rubra	L	0,2	0,3	0,0	0,2
Saxifraga sp.	F	0,0	0,1	0,4	0,2
Calluna vulgaris stem	W	0,3	0,1	0,1	0,2
Vaccinium myrtillus bud	W	0,3	0,1	0,1	0,2
Salix sp.	L	0,2	0,2	0,0	0,2
Saxifraga sp. seed	S	0,0	0,0	0,5	0,1
Saxifraga sp.	L	0,0	0,0	0,4	0,1
Cariofilaceae	L	0,2	0,2	0,1	0,1
Alchemilla plicatula	F	0,0	0,0	0,4	0,1
Saxifraga moschata stem	W	0,3	0,1	0,1	0,1
Festuca sp.	L	0,1	0,1	0,0	0,1
Dicotiledoneous flower	F	0,0	0,2	0,0	0,1
Saxifraga oppositifolia	L	0,1	0,2	0,0	0,1
Armeria/Cyperacea	F	0,3	0,0	0,1	0,1
Androsace vitaliana	L	0,1	0,1	0,1	0,1
Leucanthemopsis alpina	F	0,0	0,1	0,1	0,1
Poa sp.	L	0,0	0,0	0,2	0,1
Dryas octopetala	F	0,0	0,1	0,1	0,1
fungus	S	0,0	0,1	0,1	0,1
Thymus praecox	L	0,0	0,0	0,2	0,1
Helictotrichon sedenense	L	0,0	0,1	0,1	0,1
Asteraceae/Saxifragaceae	L	0,0	0,1	0,0	0,1
Agrostis sp.	L	0,1	0,0	0,0	0,1
Androsace carnea	L	0,0	0,0	0,2	0,1
Potentilla sp.	L	0,0	0,1	0,1	0,1
Silene acaulis	L	0,0	0,0	0,1	0,1
Polygonum vivip. (bulbils)	S	0,0	0,0	0,1	0,0
Calluna vulgaris bud	W	0,0	0,0	0,1	0,0
Calluna vulgaris flower	F	0,0	0,0	0,1	0,0
Helianthemum nummul.	L	0,0	0,0	0,1	0,0
Antennaria dioica	L	0,0	0,1	0,0	0,0
Cyperaceae seed	S	0,1	0,0	0,0	0,0
Fabaceae	L	0,0	0,0	0,0	0,0
Dryas octopetala stem	W	0,0	0,0	0,0	0,0
Dryas/Rho fer	L	0,0	0,0	0,0	0,0
Oxytropis foucadii	F	0,0	0,0	0,0	0,0
Crepis pyrenaica (bract)	F	0,0	0,0	0,0	0,0
Ericaceae	L	0,0	0,0	0,0	0,0
Saxifraga geranioides	L	0,0	0,0	0,0	0,0
Trifolium alpinum	L	0,0	0,0	0,0	0,0
Cerastium/Oxytropis	F	0,0	0,0	0,0	0,0
Luzula sp.	L	0,0	0,0	0,0	0,0
Oxytropis fruit	S	0,0	0,0	0,0	0,0
**Sample size**		**29**	**57**	**35**	**121**

S2 Table. Mean abundance (%) of food items recorded by microhistological analysis in Pyrenean rock ptarmigan faeces by year and average of three years. W woody element (stem or bud), L leaf epidermis, F floral element, S fruit, seed or fungus, A arthropod. Second and third horizontal lines mark the items that sum 80% and 90% of the total diet respectively. Sample size: n = 121; identified items = 20,301.
